# Hydrothermal Treatment of Arsenopyrite Particles with CuSO_4_ Solution

**DOI:** 10.3390/ma14237472

**Published:** 2021-12-06

**Authors:** Aleksei Kritskii, Stanislav Naboichenko

**Affiliations:** 1 Laboratory of Advanced Technologies in Non-Ferrous and Ferrous Metals Raw Materials Processing, Ural Federal University, 620002 Yekaterinburg, Russia; 2 Department of Non-Ferrous Metals Metallurgy, Ural Federal University, 620002 Yekaterinburg, Russia; elg-mtf@yandex.ru

**Keywords:** arsenopyrite, hydrothermal treatment, kinetics, mechanism, copper sulfate, sulfuric acid media

## Abstract

The nature of the hydrothermal reaction between arsenopyrite particles (FeAsS) and copper sulfate solution (CuSO_4_) was investigated in this study. The effects of temperature (443–523 K), CuSO_4_ (0.08–0.96 mol/L) and H_2_SO_4_ (0.05–0.6 mol/L) concentrations, reaction time (1–120 min), stirring speed (40–100 rpm) and particle size (10–100 μm) on the FeAsS conversion were studied. The FeAsS conversion was significant at >503 K, and it is suggested that the reaction is characterized by the formation of a thin layer of metallic copper (Cu^0^) and elemental sulfur (S^0^) around the unreacted FeAsS core. The shrinking core model (SCM) was applied for describing the process kinetics, and the rate of the overall reaction was found to be controlled by product layer diffusion, while the overall process was divided into two stages: (Stage 1: mixed chemical reaction/product layer diffusion-controlled) interaction of FeAsS with CuSO_4_ on the mineral’s surface with the formation of Cu^1+^ and Fe^2+^ sulfates, arsenous acid, S^0^, and subsequent diffusion of the reagent (Cu^2+^) and products (As^3+^ and Fe^2+^) through the gradually forming layer of Cu^0^ and molten S^0^; (Stage 2: product layer diffusion-controlled) the subsequent interaction of CuSO_4_ with FeAsS resulted in the formation of a denser and less porous Cu^0^ and S^0^ layer, which complicates the countercurrent diffusion of Cu^2+^, Cu^1+^, and Fe^2+^ across the layer to the unreacted FeAsS core. The reaction orders with respect to CuSO_4_ and H_2_SO_4_ were calculated as 0.41 and −0.45 for Stage 1 and 0.35 and −0.5 for Stage 2. The apparent activation energies of 91.67 and 56.69 kJ/mol were obtained for Stages 1 and 2, respectively.

## 1. Introduction

Arsenopyrite (FeAsS) is the most common arsenic (As)-containing mineral among ore sulfide deposits, and it is also of limited economic importance and is generally discarded as a solid waste during the mining operation [1]. If FeAsS present in an ore/concentrate is associated with significant gold values, then the material is typically hydrometallurgically treated, since the conventional roasting leads to the release of As into the environment [2,3,4,5,6,7,8]. The most common approach is subjecting FeAsS ore/concentrate to pressure oxidative leaching in order to release precious metals from the mineral’s crystal lattice and at the same time to isolate As in the form of hardly soluble scorodite (FeAsO_4_·5H_2_O) [9,10,11]. Otherwise, the presence of FeAsS, as well as other As-containing minerals in copper concentrates, leads to the contamination of refined copper [12] and environmental pollution [13]. Moreover, As accumulates in copper flue dust, complicating the processing of the latter [14]. Thus, existing copper smelters prefer to receive concentrates that are almost free of toxic elements [15].

The hydrothermal treatment of copper concentrates with CuSO_4_ solution in acidified media (H_2_SO_4_) is currently an area of interest since it allows for enriching concentrates with copper content and removing iron [16,17,18,19]. The enrichment of concentrates is achieved by transforming chalcopyrite (CuFeS_2_) into secondary copper sulfides (CuS, Cu_1.8_S, Cu_1.94_S and Cu_2_S) through the exchange reactions between copper sulfate (CuSO_4_) and CuFeS_2_ [20,21,22,23,24]. Another advantage of the process is the simultaneous purification of the concentrates from a number of impurities; the latter is achieved by the interaction of accompanied sulfide minerals (ZnS, PbS, FeS_2_, FeAsS, MoS_2_, etc.) with CuSO_4_ solution [20,24,25,26,27,28]. While the behavior of the most common impurities (ZnS, FeS_2_, PbS) is well discussed in the literature [20,28], As-containing minerals have not received wide attention, although their behavior is of key importance in copper metallurgy.

In 2019, Fuentes [29] proposed the hydrothermal treatment of Chilean copper concentrates with a significant arsenic content at temperatures up to 573 K in H_2_SO_4_ media. Such a high-temperature treatment allowed for the transfer of more than 90% As, predominantly present in the concentrate as enargite, into the solution, thus producing high-quality copper concentrate. However, the kinetic characteristics of the reaction were not given. As for FeAsS, any information on its behavior during hydrothermal treatment with CuSO_4_ solution is not available in open sources.

For comparison reasons, the kinetics of FeS_2_ hydrothermal treatment with CuSO_4_ solution is briefly reviewed, since both FeAsS and FeS_2_ are considered as refractory to hydrometallurgical treatment and often present together in sulfide ores. Hydrothermal treatment of FeS_2_ has been found to be chemically controlled [20], exhibiting fractional order dependencies with respect to CuSO_4_ and H_2_SO_4_ [20,27]. FeS_2_ conversion becomes significant at temperatures higher than 503 K. Activation energy has been calculated as 108 kJ/mol. The surface of the mineral after hydrothermal treatment was detected to be covered by a multilayer film of copper sulfides (Cu_1.8_S, Cu_2_S). Despite the comprehensive literacy of the mentioned works, the kinetic analysis was not conducted at the very beginning of the process—more emphasis was placed on a longer duration (0.5–4 h), which could lead to the omission of important dependencies in the development of the process. The FeS_2_ hydrothermal treatment kinetics was investigated in slightly acidified solutions (H_2_SO_4_, pH 1.3–1.4).

The current work presents a kinetic study on the hydrothermal treatment of FeAsS particles with CuSO_4_ solution. The effects of temperature (443–523 K), CuSO_4_ (0.08–0.96 mol/L) and H_2_SO_4_ (0.05–0.6 mol/L) concentrations in the initial solution, particle size (10–100 μm), and stirring speed (40–100 rpm) on FeAsS conversion were investigated to find the optimal conditions. A shrinking core model (SCM) was used to describe the kinetics of the process. A mechanism of the interaction is proposed. Research data could be used for the industrial process design.

## 2. Materials and Methods

### 2.1. Methodology

**Figure 1:**
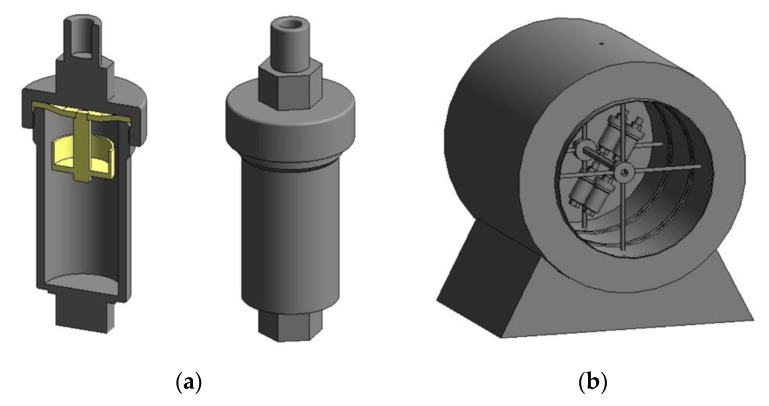
Experimental setup on investigation of hydrothermal treatment process: (**a**) titanium reactor and (**b**) cylindrical furnace.

The experiments were performed in a laboratory setup, simulating conditions for autoclave hydrothermal interaction processes (Figure 1). The experimental set-up was a cylindrical furnace located in a horizontal plane. A door was mounted in one of the sidewalls of the furnace for fastening and removing sealed titanium reactors on a rotating shaft. A hole for the rotating shaft and its output to the engine is located on the opposite sidewall (Figure 1b). The titanium reactors (45 mL) consist of two parts—a reactor and a lid. The reactor and the lid are interconnected by a standard spiral thread and sealed using a fluoroplastic gasket. An additional hole was made in the center of the gasket to fasten the baskets with the material inside (it limits the interaction of the material with the solution until the required temperature is reached) (Figure 1a). Mixing was achieved by rotating the shaft on which the reactors are mounted. Temperature was measured with a thermocouple, which was placed inside the furnace through the hole at the top.

In all the experiments, a sample of 0.2 g FeAsS was put into a basket and a portion of 30 cm^3^ solution with required concentration of CuSO_4_ and H_2_SO_4_ was poured into the reactor; the reactor was sealed and fasten to the shaft. The cylindrical furnace was heated up to the desired temperature and rotation of the shaft was turned on—this moment was considered as the beginning of the experiment. Neither additional reacting gases were introduced into the reactors during the experiments, and the overall pressure in the reactor was equal to the vapor pressure of water at the appropriate temperature.

The mineralogical and chemical compositions of the mineral and solid residues were determined based on the detailed optical and scanning electron optical microscopy coupled with energy dispersive spectroscopy “SEM-EDS” (Carl Zeiss Sigma VP, ZEISS Microscopy, Oberkochen, Germany), energy dispersive X-ray fluorescence spectrometry “XRF” (Shimadzu EDX-7000), X-ray diffraction “XRD” (XRD-7000, Shimadzu Corp., Japan), wet analysis using inductively coupled plasma atomic emission spectroscopy “ICP-ES” (iCAP 6500 Duo, Thermo Electron Corporation, Waltham, MS, USA) and laser diffraction (Helos/BR, Sympatec, Clausthal-Zellerfeld, Germany). For SEM-EDS scanning, the molds (hot pressing) with the samples from conductive materials were made and were subsequently subjected to accurate grinding. The solid materials were dissolved in aqua regia before subjecting to ICP-ES. The sulfur content was analyzed using carbon/sulfur analyzer (CS 230, LECO Corp., St. Joe, MO, USA). Solutions composition was analyzed by ICP-ES; concentration of H_2_SO_4_, Fe^2+^, As^3+^ was analyzed by titration.

The FeAsS conversion (E, %) and fraction reacted (X) were calculated according to the following Equations (1) and (2), respectively:

(1) $$E=\frac{m_{s}}{m_{i}}\cdot 100$$

(2) $$X=\frac{m_{s}}{m_{i}}\;$$

where m_s_ and m_i_ are the mass of As (or Fe) in solution after the treatment and initial FeAsS, respectively.

### 2.2. Materials and Characterization

A high purity specimen of FeAsS mineral originating from Beryozovskoe deposit (Beryozovsky, Sverdlovsk oblast, Russian Federation) was used in this study.

**Figure 2:**
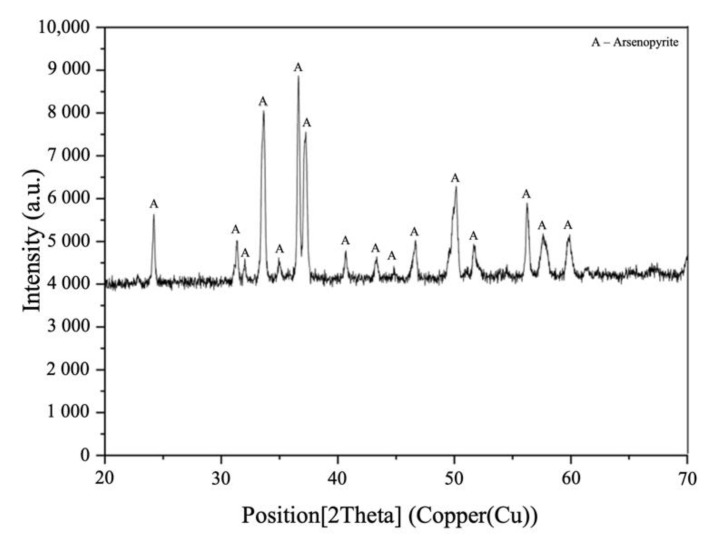
XRD pattern of the initial FeAsS particles (10–29 μm).

The samples for autoclave experiments were obtained from the ground crystals by wet sieving. According to ICP-ES analysis, FeAsS has the following chemical composition, by percentages: 33.6 Fe, 45.2 As, 18.9 S. No significant amounts of other sulfide components were detected (Figure 2), and insignificant presence of quartz (SiO_2_) is possible.

The particle size analysis of the ground mineral is shown in Table 1.

## 3. Results and Discussion

### 3.1. Discussion Details

The current study was aimed at optimizing the hydrothermal treatment parameters to achieve a higher FeAsS particles conversion in sulfuric acid media using CuSO_4_ as an oxidant. In Section 3.1.1, Section 3.1.2, Section 3.1.3, Section 3.1.4 and Section 3.1.5, the influence of temperature (443–523 K), CuSO_4_ (0.08–0.96 mol/L) and H_2_SO_4_ (0.05–0.6 mol/L) concentrations in the initial solution, particle size (10–100 μm) and stirring speed (40–100 rpm) on the FeAsS particles’ conversion was studied to determine the most significant factors. The final solution (Section 3.1.6) and the solid residue (Section 3.2) compositions were analyzed to identify the reaction products and to suggest the probable chemical reactions of the interaction. Finally, the kinetics of the process was analyzed (Section 3.3), and kinetics equations were established (Section 3.4) that suggested a probable mechanism of the interaction.

#### 3.1.1. Effect of Stirring Speed

**Figure 3:**
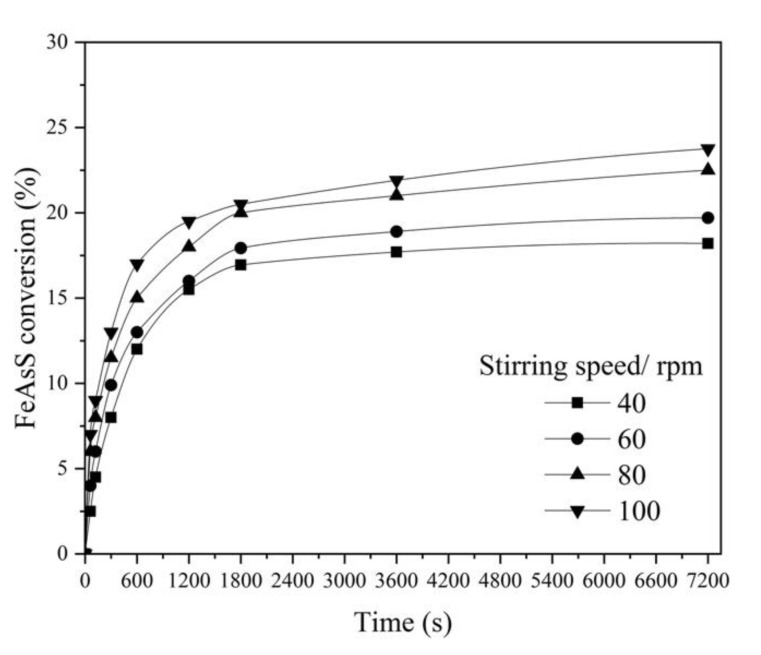
Effect of stirring speed on hydrothermal conversion of FeAsS (503 K; 0.1 mol/L of H_2_SO_4_; 0.16 mol/L of Cu; 10–29 μm).

The effects of stirring speed, temperature and particle size were studied with 0.16 mol/L of Cu, which is sufficient for the stoichiometric reaction Equation (3); the standard concentration of H_2_SO_4_ was established as 0.1 mol/L, since the autoclave treatment of sulfide materials most often carried out in an acidic media (H_2_SO_4_, pH 1–2) due to the oxidation of sulfur to sulfate [30]. An increase in stirring speed has a positive effect on FeAsS conversion (Figure 3). After 7200 s of reaction at 100 rpm, the conversion exceeded 23%.

Although the kinetics research technique in leaching recommends excluding the external diffusion by increasing the stirring speed until the positive effect on the conversion rate is neutralized, an excessive increase in the stirring speed in the present equipment (Figure 1) can lead to the formation of a stagnant zone. Thus, all subsequent experiments were conducted at 100 rpm in order to investigate the effect of other factors.

According to the figure, two stages of the reaction progress were also observed: during the first 600 s of the process (Stage 1), the reaction rate was more than 10 times higher than at the following period 1200–7200 s (Stage 2).

The parabolic shape of the kinetic curves (Figure 3) suggests that the reaction rate is controlled by product layer diffusion [31,32,33] due to the formation of a product layer on the surface of the unreacted core of FeAsS particles.

#### 3.1.2. Effect of Temperature

**Figure 4:**
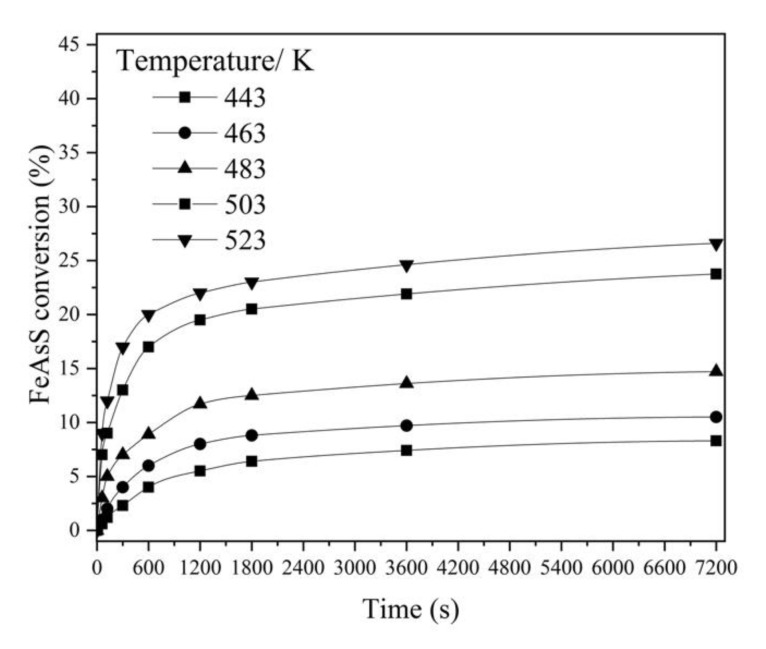
Effect of temperature on hydrothermal conversion of FeAsS (100 rpm; 0.1 mol/L of H_2_SO_4_; 0.16 mol/L of Cu; 10–29 μm).

In this study, a high temperature range was chosen based on a kinetics study on FeAsS oxidation in autoclave published in [10]. The hydrothermal treatment results obtained at different temperatures (443–523 K) are shown in Figure 4. The increase in temperature significantly affects FeAsS conversion; at T = 443 K for 7200 s, only 7% of FeAsS was reacted, while at 523 K, conversion increased by more than three times with the same process time. The two-stage reaction progress was again observed in the similar duration intervals. Since higher temperature damages the connecting carving of the reactor, all subsequent experiments were conducted at 503 K.

FeAsS requires a high temperature treatment for significant conversion. This is also true for other sulfide minerals such as ZnS (>453 K) [28], FeS_2_ (>473 K) [20] and CuFeS_2_ (>453 K) [16].

#### 3.1.3. Effect of H_2_SO_4_ Concentration

**Figure 5:**
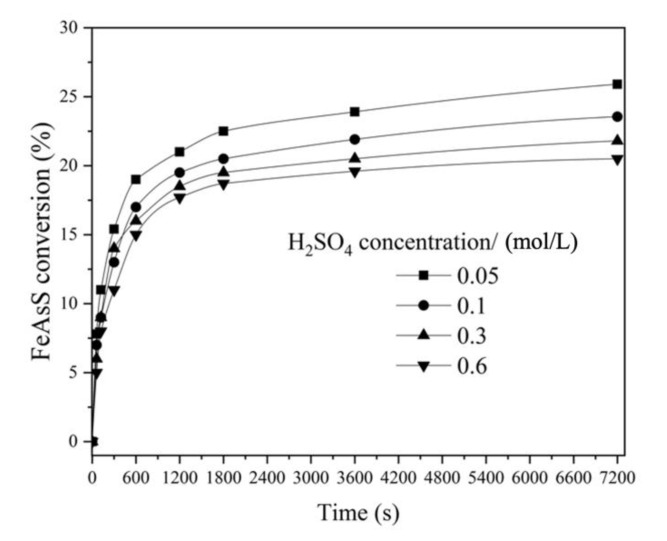
Effect of H_2_SO_4_ concentration on hydrothermal conversion of FeAsS (100 rpm; 503 K; 0.16 mol/L of Cu; 10–29 μm).

The effect of H_2_SO_4_ concentration ranging from 0.05 to 0.6 mol/L on FeAsS conversion was investigated. The results in Figure 5 show a moderate decrease in reaction rate with the increase in H_2_SO_4_ concentration. After 7200 s of reaction at 0.6 mol/L of H_2_SO_4_, about 20% of FeAsS was reacted, while at 0.05 mol/L, conversion increased up to 25%.

A moderate deceleration in reaction rate with the H_2_SO_4_ concentration increase was also found for the hydrothermal treatment of CuFeS_2_, FeS_2_ and ZnS [21,25,26], where such a dependency indicates a sulfuric acid formation as a result of the interaction. Thereby, it is proposed the interaction proceeds according to Equation (3):

(3) $$2.5{\mathrm{CuSO}}_{4}{+\mathrm{FeAsS}+2H}_{2}{O=\mathrm{FeSO}}_{4}{+\mathrm{HAsO}}_{2\;}+2.5{\mathrm{Cu}+S}^{0}+1.5H_{2}{\mathrm{SO}}_{4};\;(\Delta G=-93\;\mathrm{kJ};\;498\;K)\;$$

All the experiments were conducted at 0.1 mol/L of H_2_SO_4_, since the hydrothermal treatment of sulfides is performed in conditions of H_2_SO_4_ formation at pH = 1 [17,20].

#### 3.1.4. Effect of CuSO_4_ Concentration

**Figure 6:**
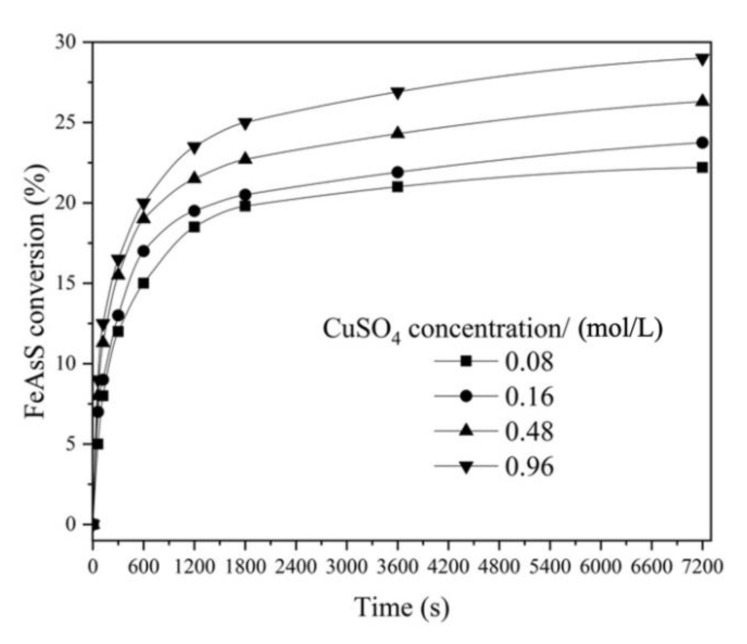
Effect of CuSO_4_ concentration on hydrothermal conversion of FeAsS (100 rpm; 503 K; 0.1 mol/L of H_2_SO_4_; 10–29 μm).

The effect of CuSO_4_ concentration ranging from 0.16 to 0.96 mol/L on FeAsS conversion was investigated. Figure 6 shows the results of a moderate increase in reaction rate with the increase in CuSO_4_ concentration. During 7200 s, the FeAsS conversion increased from 23 to 28% at 0.16 and 0.96 mol/L, respectively.

The insignificant effect of CuSO_4_ as well as that of H_2_SO_4_ on the reaction rate may also indicate that the process is controlled by diffusion through the product layer. Similar dependencies have been reported for the hydrothermal treatment of ZnS [19], CuFeS_2_ [23] and Cu_5_FeS_4_ [20].

#### 3.1.5. Effect of FeAsS Particle Size

**Figure 7:**
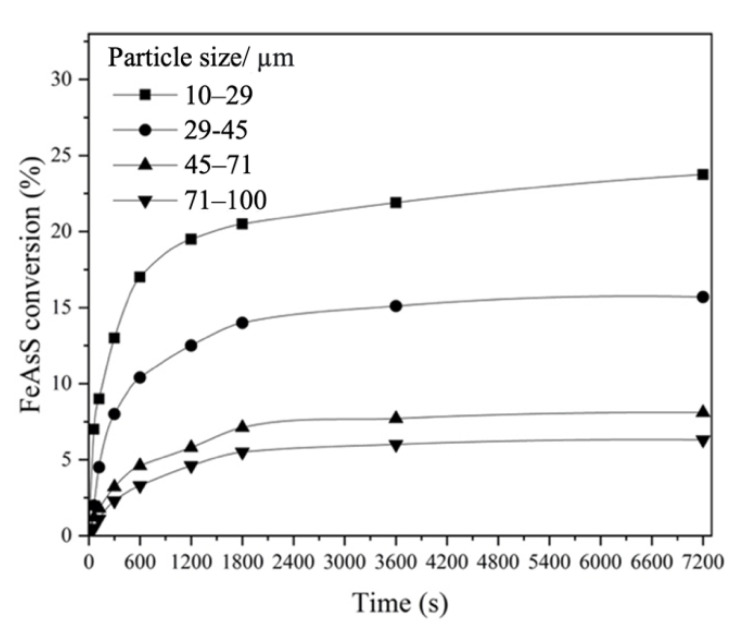
Effect of FeAsS particle size on its hydrothermal conversion (100 rpm; 503 K; 0.1 mol/L of H_2_SO_4_; 0.16 mol/L of Cu).

Four particle sizes (10–29 μm, 29–45 μm, 45–71 μm, 71–100 μm) were used in the experiments. The results are shown in Figure 7. As expected, a smaller particle size resulted in higher FeAsS conversion. With the decrease in particle size, the specific surface area increases, and the internal diffusion resistance decreases, accelerating the reaction. In experiments with the particle size of 74–100 μm, FeAsS conversion slightly exceeded 6%, while in experiments with the particle size of 10–29 μm, the conversion increased by more than three times and reached 23.75%.

Significant conversion rate dependency on the particle size is an additional indication that the kinetics may be controlled by diffusion through the product layer [31,32]. A similar effect of the particle size was observed in [19,23].

#### 3.1.6. Behavior of Iron in Hydrothermal Interaction of FeAsS with CuSO_4_

Additional analysis was performed to study the behavior of As and Fe during the hydrothermal treatment of FeAsS.

**Figure 8:**
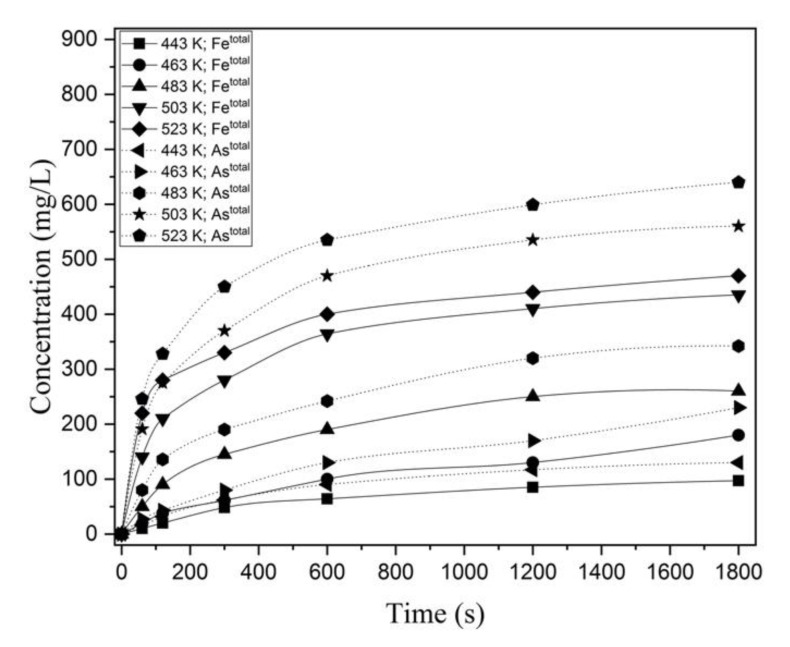
Concentration of As and Fe as a function of the process time at different temperatures (443–523 K; 100 rpm, 0.1 mol/L of H_2_SO_4_; 0.16 mol/L of Cu; 10–29 μm).

Figure 8 shows that Fe transfers into the solution at a similar ratio with As. According to redox titration with KBrO_3_ and KMnO_4_ solutions (respectively for As^3+^ and Fe^2+^), As and Fe are predominantly present in the solution in trivalent and bivalent forms, respectively, which suggests the formation of arsenic acid and ferrous sulfate as the reaction products.

### 3.2. Characterization of Residue

**Figure 9:**
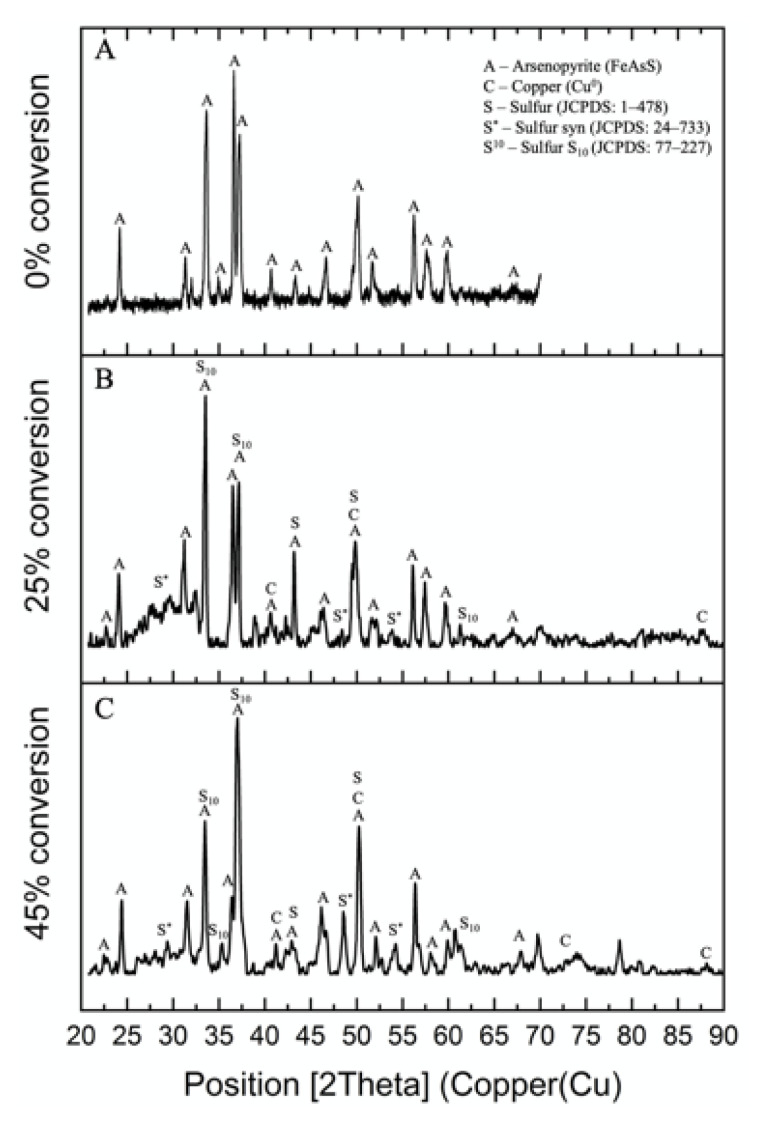
XRD pattern of FeAsS residue at different conversion degrees: (**A**) 0% conversion; (**B**) 25% conversion; (**C**) 45% conversion).

Figure 9 shows the XRD patterns of solid residue after hydrothermal treatment at different FeAsS conversion degrees. According to the figure, it is difficult to accurately conclude the reaction product form; however, along with conversion progress, there was a noticeable increase in the intensity of some of the FeAsS peaks that match metallic copper (Cu^0^) and varied sulfur allotropes (S^0^) [34] peaks (Figure 9B,C).

In addition, the presence of S^0^ was confirmed by leaching the residue in a solution of sodium sulfide in an alkaline medium. Table 2 shows the chemical composition of the residue before and after treatment in sodium sulfide solution.

In the context of the hydrothermal treatment of FeAsS with CuSO_4_ solution, a layer of Cu^0^ and S^0^ is suggested to form a diffusion barrier according to Equation (3), which prevents the reactants from coming into contact with the unreacted core. Diffusion across the product layer is mainly dependent on the thickness and porosity of the layer. In fact, the possibility of reacting in the internal diffusion zone depends firstly on the density of the product layer [31,32]. The higher the density is, the smaller the porosity, and the more difficult it is for the reactant and liquid products to flow across the product layer. The density of the products layer can usually be measured by the value of Z or the Pilling–Bedworth ratio, as seen in Equation (4):

(4) $$K_{P-B}=\frac{{c\cdot V}_{\mathrm{product}}}{{a\cdot V}_{\mathrm{reactant}}}=\frac{c\cdot \frac{M_{\mathrm{product}}}{p_{\mathrm{product}}}}{a\cdot \frac{M_{\mathrm{reactant}}}{p_{\mathrm{reactant}}}}$$

where c/a is the number of moles of solid product formed by one mole solid reactant; M_product_ is the molar weight of the solid product (Cu^0^ or S^0^), 64 or 32 g/mol; p_product_ is the density value of Cu^0^ or S^0^, 8.96 or 2 g/cc; M_reactant_ is the molar weight of the solid reactant (FeAsS), 163 g/mol; and p_reactant_ is the density value of FeAsS, 6 g/cc. In the context of the joint Cu^0^ and molted S^0^ presence on the surface of FeAsS, Z = 1.25 means that a product layer could form a diffusion barrier.

**Figure 10:**
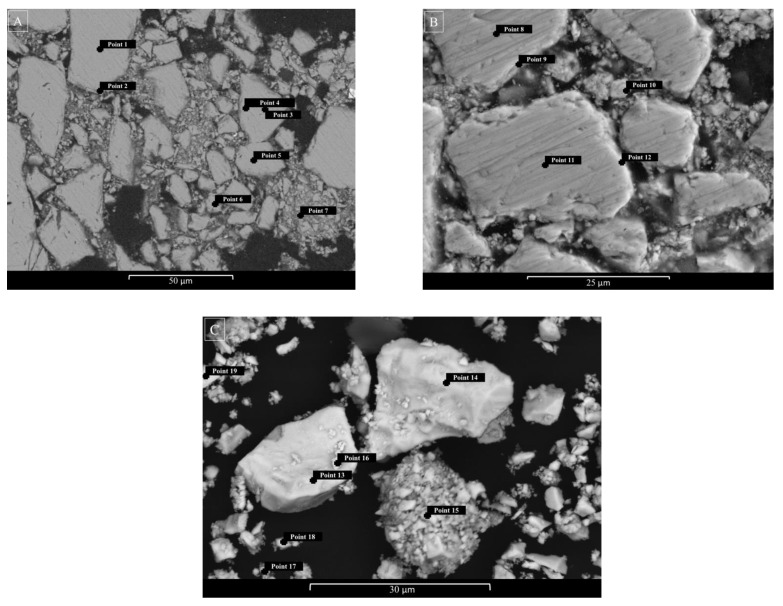
SEM images of the initial FeAsS particles (**A**) and particles after hydrothermal treatment (**B**) in cross-sectional view; bulk particles (**C**) after hydrothermal treatment.

To confirm the conclusions on the nature of the process mentioned above, SEM–EDS scanning (EHT = 20 kV) in BSE (back-scattered electrons) and/or SE (secondary electrons) regimes were performed for the microstructure investigation of FeAsS particles before and after hydrothermal treatment (523 K, 100 rpm, 0.1 mol/L of H_2_SO_4_, 0.16 mol/L of Cu, 10–29 μm). These results are shown in the Figure 10A–C.

Table 3 shows the chemical composition of the particles at the points indicated in Figure 10. SEM scanning in the BSE/SE regime cannot visually determine a clear boundary between the surface film and the unreacted core, although the results of chemical analysis at Points 9, 10 and 12 (Table 3, Figure 10B) distinctly indicate the presence of copper and an increase in sulfur content. The EDS analysis of the bulk particles after hydrothermal treatment (Table 3, Figure 10C) also indicates that the surface of the particles becomes enriched with sulfur and contains copper.

**Figure 11:**
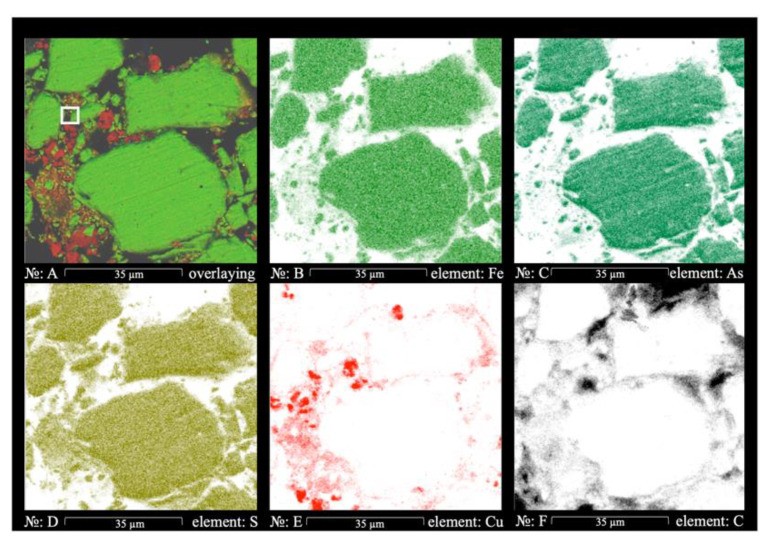
EDS mapping of FeAsS particles after hydrothermal treatment at 523 K, 100 rpm, 0.1 mol/L of H_2_SO_4_, 0.16 mol/L of Cu, 10–29 μm in cross-sectional view: overlaying (**A**); As distribution (**B**); Fe distribution (**C**); S distribution (**D**); Cu distribution (**E**); C distribution (**F**).

Figure 11 shows the multilayer EDS mapping of the residue. According to the figure, copper (Figure 11E) is present on the surface of FeAsS particles and as clots. Figure 11D show that clots are almost free of sulfur, which confirms that copper is present in the residue as Cu^0^. Regarding the nature of the clots’ formation, it seems that part of the Cu (1+) diffuses through the layer of elemental sulfur as sulfate and disproportionates in the solution, resulting in Cu^0^ spreading throughout the residue in the form of free particles.

**Figure 12:**
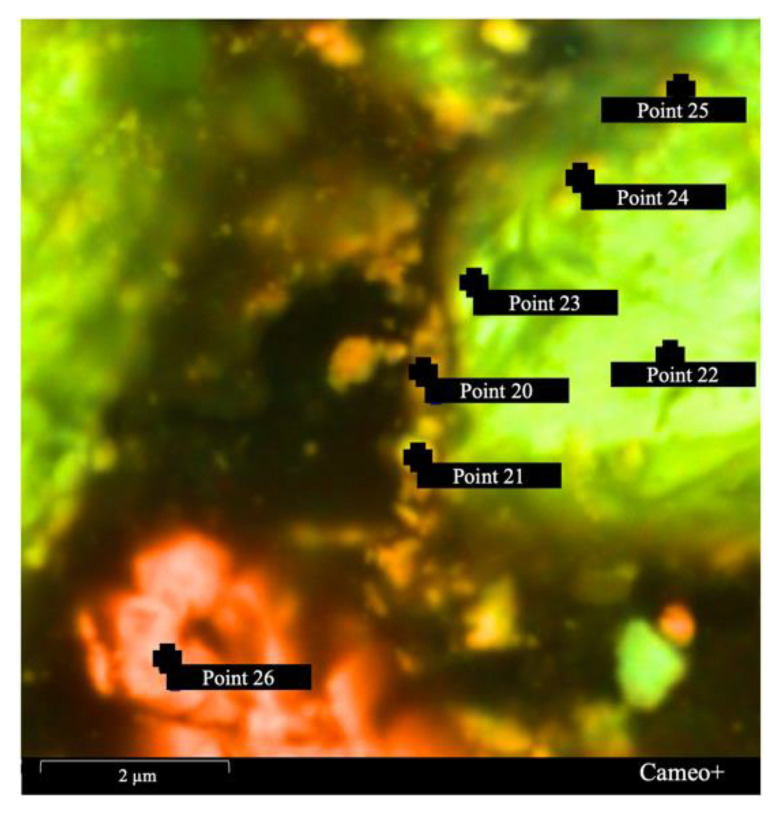
SEM image of the sector, shown in Figure 11A.

In order to more accurately identify the chemical composition of the boundary surface on FeAsS particles after treatment, the sample was analyzed at high magnification. Figure 12 shows the SEM image of the sector, determined in Figure 11A. The chemical composition of the surface boundary at the points indicated in Figure 12 are shown in Table 3. Therefore, the SEM-EDS analysis additionally confirmed the assumption that the mineral surface is covered by film consisting of Cu^0^ and S^0^.

It is worth mentioning that according to SEM-EDS, some of the copper is associated with oxygen, which is quite expected due to the fact that during preparation, the samples for the microscopic examination of Cu^0^ could have been partially oxidized. Oxygen was identified during the SEM-EDS analysis, especially during the creation of EDS maps (Figure 10, Figure 11 and Figure 12), but it was excluded due to its insignificant content (1–7%).

### 3.3. Hydrothermal Treatment Kinetics

Thus, it is appropriate to conclude that the rate controlling step of the overall reaction is diffusion, and the reaction proceeds in two stages: (Stage 1: mixed chemical reaction/product layer diffusion-controlled) interaction of FeAsS with CuSO_4_ on the mineral’s surface with the formation of Cu(1+) and Fe(2+) sulfates, arsenic acid, S^0^ and the subsequent diffusion of the reagent (Cu^2+^) and products (As^3+^ and Fe^2+^) through the gradually forming layer of Cu^0^ and S^0^; (Stage 2: product layer diffusion-controlled) the subsequent interaction of CuSO_4_ with the FeAsS, resulting in the formation of a denser and less porous Cu^0^ and S^0^ layer, which complicates countercurrent diffusion of Cu^2+^, Cu^1+^ and Fe^2+^ across the layer to the unreacted FeAsS core.

According to the analysis of the kinetic curves and the microstructure of the material, it is appropriate to perform a kinetic description of the process using the shrinking core model (SCM). Table 4 presents kinetics equations that were applied to describe the liquid–solid reaction [31,35,36]

According to Equations (A)–(C) in Table 4, the function of time “*t*” should be represented by a straight line with the slope angle “k”. For the kinetic analysis, the SCM equations from Table 4 were applied to the experimental data on the hydrothermal treatment of FeAsS with CuSO_4_ solution at *t* = 443–523 K (100 rpm; 0.1 mol/L of H_2_SO_4_; 0.16 mol/L of Cu); the correlation coefficient (R^2^) determines the standard deviation of the experimental data from a straight line (Table 5).

As can be seen from data obtained, none of the SCM Equations (A)–(C) (Table 5) can be applied to describe the hydrothermal process, since the correlation coefficient is less than 0.9 and even shows negative values.

**Figure 13:**
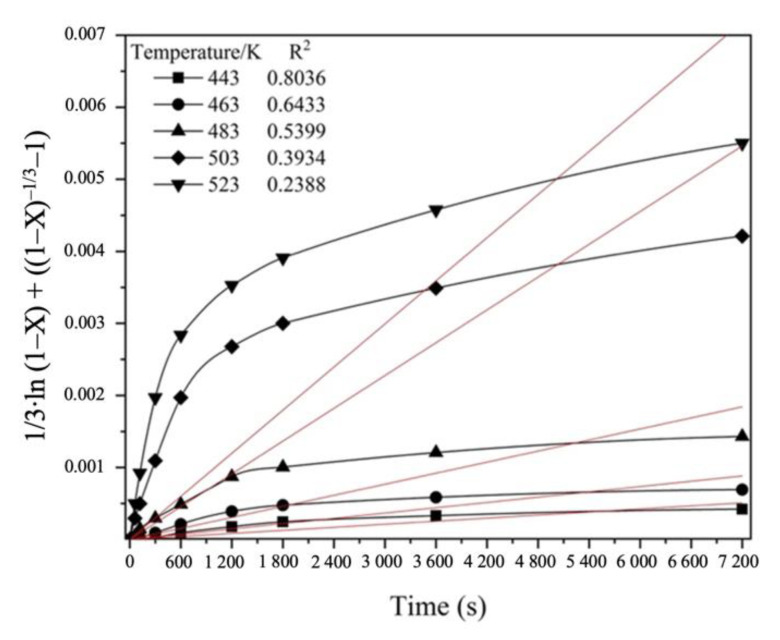
Linear relationship between 1/3 · ln (1 − X) + ((1 − X)^−1/3^ − 1) and hydrothermal treatment time at various temperatures.

Additionally, the results for linear approximation between hydrothermal treatment time and the “new shrinking core model” kinetics equation are shown in Figure 13.

Therefore, the current process cannot be described by known kinetics equations, since it consists of two different stages, as previously mentioned: (Stage 1) 0–600 s kinetics is controlled by a mixed chemical reaction (the chemical interaction of FeAsS with CuSO_4_ on the FeAsS surface) and diffusion through the primary product layer (diffusion of CuSO_4_ across the Cu^0^-S^0^ layer) control; (Stage 2) 1200–7200 s kinetics is controlled by the diffusion through the product layer (the diffusion of CuSO_4_ across the condensed Cu^0^-S^0^ layer to the unreacted FeAsS core).

**Figure 14:**
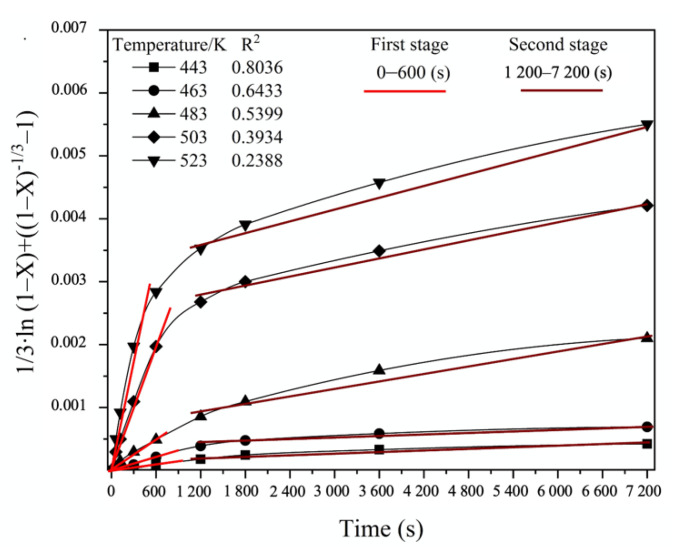
Defined stages on the plot of linear relationship between $1/3\cdot \ln\;(1-X)+{((1-X)}^{-1/3}-1)$ and hydrothermal treatment time.

On the contrary, the hydrothermal process of FeAsS treatment can be described by two separate kinetics equations at corresponding stages. In Figure 14, defined stages that show straightness on an approximation plot in accordance with the “new shrinking core model” are shown.

**Figure 15:**
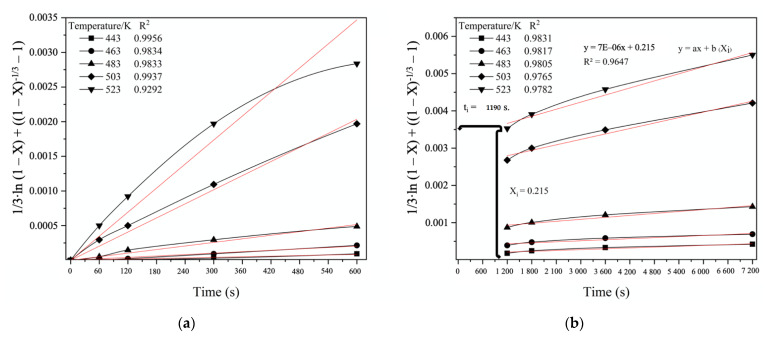
Linear relationship between $1/3\cdot \ln\;(1-X)+{((1-X)}^{-1/3}-1)$ and hydrothermal treatment time at defined stages and various temperatures. Stage 1: 0–600 s (**a**); Stage 2: 1200–7200 s (**b**).

Figure 15 shows the linear relationship between the “new shrinking core model” Equation and Stage 1 (Figure 15a) and Stage 2 (Figure 15b) of the FeAsS treatment. The process interval 600–1200 s is characterized by the transition from Stage 1 to Stage 2.

As can be seen from the results of the linear approximation fitting, the kinetics data mostly correspond to Equation (B) (Table 6), which is suggested to be applied to describe the hydrothermal process of FeAsS treatment, since the R^2^ coefficient is higher than the other equations show.

**Figure 16:**
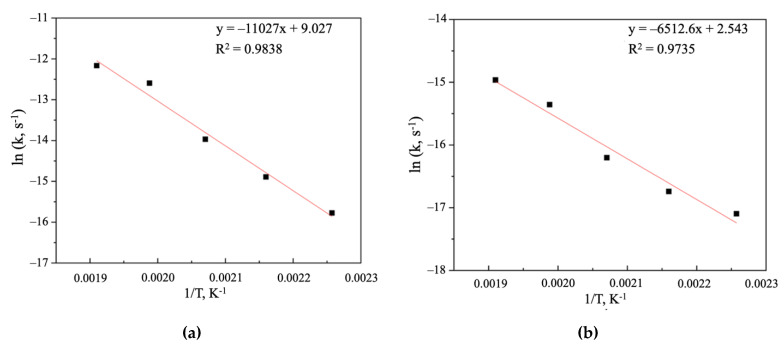
Arrhenius plot for Stage 1 (**a**) and Stage 2 (**b**).

The apparent reaction rate constant (*k*) at temperatures of 443, 463, 483, 503 and 523 K, respectively, was 1.5829 × 10^−7^, 3.7063 × 10^−7^, 8.0899 × 10^−7^, 3.2132 × 10^−6^ and 4.6362 × 10^−6^ (s^−1^) for Stage 1 and 8.4044 × 10^−8^, 7.5062 × 10^−7^, 1.1829 × 10^−7^, 2.4405 × 10^−7^ and 3.5083 × 10^−6^ (s^−1^) for Stage 2. Figure 16 shows the Arrhenius plots, where the coefficient “a” in the equation “y = ax + b” is equal to −11027 for Stage 1 (Figure 15a) and −6512.6 for Stage 2 (Figure 15b), which is in accordance with the Arrhenius law, allowing us to calculate the activation energy—91.67 and 56.69 kJ/mol. Therefore, a high activation energy value for Stage 1 confirms the assumption that the kinetics of the stage is controlled by mixed chemical reaction/diffusion throughout the product layer and, correspondingly, lower activation energy on the Stage 2 confirms that the process is controlled by diffusion throughout the product layer [31,32]. Although the activation energy seems to suggest a chemical reaction control, recent studies have shown that some diffusion-controlled reactions have unusually high activation energy [37,38,39]. Moreover, the hydrothermal processes of sulfide minerals treatment with copper sulfate solutions in most cases are characterized by high activation energies [20,23,24,32] and in the case of sphalerite, chalcopyrite and bornite, the diffusion-controlled kinetics of the process was concluded.

The reaction order with respect to CuSO_4_ and H_2_SO_4_ was calculated as 0.41 and −0.45 for Stage 1 and as 0.35 and −0.5 (Figure 5 and Figure 6) for Stage 2. The fractional order with respect to the reagents is also typical for hydrothermal processes mentioned above.

The rate-controlling step of the process can also be identified from the temperature coefficient of the reaction speed. For the diffusion-controlled process, the temperature coefficient is generally 1.3–1.6, while for the chemical reaction control process, the temperature coefficient is ≥ 2. The experimental results in Figure 15 show that in the process of the reaction temperature rising from 443 to 523 K with the temperature step 20 K, the average temperature coefficient is 1.7 for Stage 1 (Figure 15a) and 1.6 for Stage 2 (Figure 15b), which corresponds to the diffusional control process.

According to the balance experiments, the process of FeAsS hydrothermal treatment with CuSO_4_ solution is accompanied by the formation of H_2_SO_4_. As for stoichiometry, 1 mole of Fe (2+) and As (3+), 2–3 moles of Cu^0^, 1–2 moles of H_2_SO_4_ and 0.5–1.5 moles S^0^ are formed per mole of FeAsS. Thus, it is proposed that this process is described by reaction 3.

### 3.4. Establishment of the Kinetic Equations

Although the general kinetic equation for FeAsS treatment with CuSO_4_ solution cannot be determined, the process can be divided into two stages, and it is suggested that each stage is described by individual kinetics equation. The kinetics equations of the total apparent reaction rate constant were determined according to the above-mentioned results, considering the effects of the initial concentration of CuSO_4_ and H_2_SO_4_ and reaction temperature. The rate expression for this hydrothermal process can be written as follows in Equation (5):

(5) $$1/3\cdot \ln\;(1-X)+((1-X{)}^{-1/3}-{\;1)=k}_{0}{\cdot C}_{1}{\cdot C}_{2}{\cdot D}_{0}\cdot \exp[-E_{d}/(R\cdot T)]\cdot t$$

where C_1_ and C_2_ are reaction orders with respect to CuSO_4_ and H_2_SO_4_, respectively; D_0_ is the pre-exponential factor of the diffusion coefficient expressed as the Arrhenius-type equation; E_d_ is the activation energy; T is the temperature; R is the gas constant; and *t* is the reaction time.

**Figure 17:**
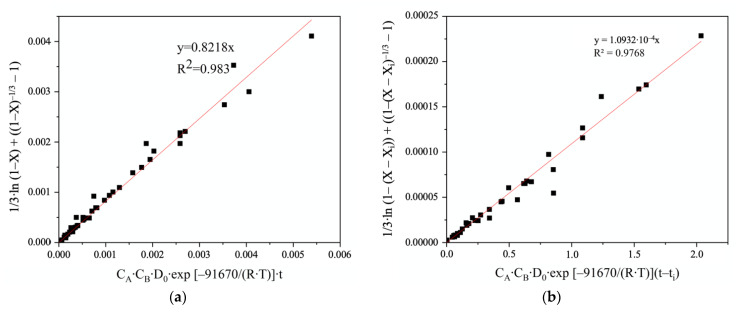
Relationship between SCM equation and k_0_·C_1_·C_2_·D_0_·exp [−E_d_/(R·T)]·*t* in the hydrothermal treatment of FeAsS process for Stage 1 (**a**) and Stage 2 (**b**), respectively.

The experimental data obtained at different conditions were substituted into Equation (5) for Stage 1 of the process. Figure 17a shows that the relationship between $1/3\cdot \ln\;(1-X)+({\left(1-X\right)}^{-1/3}-1)$ and $k_{0}{\cdot C}_{1}{\cdot C}_{2}{\cdot D}_{0}\cdot \exp\;[-E_{d}/(R\cdot T)]\cdot t$ for all experimental data was established, and the data points were mostly distributed around a line with the linear correlation coefficient of R^2^ = 0.983.

For describing the Stage 2 in a similar way (Figure 17b), non-dimensional variables (*X_i_* and *t_i_*, respectively) were introduced into $1/3\cdot \ln\;(1-X)+{((1-X)}^{-1/3}-1)$ and $k_{0}{\cdot C}_{1}{\cdot C}_{2}{\cdot D}_{0}\cdot \exp\;[-E_{d}/(R\cdot T)]\cdot t$ equations, which allowed the kinetic curves of Stage 2 (Figure 15b) to shift to the beginning of axe.

According to the reaction orders apparent activation energies, the kinetic equations of FeAsS hydrothermal treatment with CuSO_4_ solution for Stage 1 and Stage 2 can be expressed as Equations (6) and (7):

(6) $$1/3\cdot \ln\;{(1-X)}^{-1/3}-1)=0.8218\cdot {\left[{\mathrm{CuSO}}_{4}\right]}^{0.41}{\cdot [H}_{2}{\mathrm{SO}}_{4}{]}^{-0.45}{\cdot D}_{0}\cdot \exp[-91670/(8.314\;T)]\cdot t$$

(7) $$1/3\cdot \ln\;(1-(X-X_{i})+((1-(X-X_{i}{)}^{-1/3}-\;1)=0.0082\cdot [{\mathrm{CuSO}}_{4}{]}^{0.35}{\cdot [H}_{2}{\mathrm{SO}}_{4}{]}^{-0.5}{\cdot D}_{0}\cdot \exp[-56692/(8.314\cdot T)]\cdot (t-t_{i})\;$$

where, for Stage 1, Equation (6) is applicable for the interval 0 < *t* ≤ 600; for Stage 2, Equation (7) is applicable for the interval 1200 < *t* ≤ 7200.

## 4. Conclusions

The effects of stirring speed, temperature, CuSO_4_ and H_2_SO_4_ concentrations and particle size on FeAsS particles’ conversion were analyzed to suggest a mechanism of the hydrothermal process. The results indicate that temperature and FeAsS particle size have the significant influence on the reaction rate; FeAsS conversion was significant at T > 483 K. SEM-EDS analysis of the solid residue after the treatment confirmed that the product layer consisting of Cu^0^ and S^0^ was formed during the reaction. It was found that the overall reaction proceeds in two stages:

1. (Stage 1) 0–600 s kinetics is controlled by mixed chemical reaction (chemical interaction of FeAsS with CuSO_4_ on the FeAsS surface) and diffusion (diffusion of CuSO_4_ across the primary Cu^0^-S^0^ layer);
2. (Stage 2) 1200–7200 s kinetics is controlled by diffusion through the product layer (diffusion of CuSO_4_ across the condensed Cu^0^-S^0^ layer to unreacted FeAsS core).

The apparent activation energies for Stage 1 and Stage 2 were calculated as 91.67 and 56.69 kJ/mol, respectively. The reaction orders with respect to CuSO_4_ and H_2_SO_4_ were calculated as 0.41 and −0.45 for Stage 1 and 0.35 and −0.5 for Stage 2, respectively. The kinetics data were summarized in a form of kinetics equations for each stage of the process, separately.

Summing up, it is appropriate to conclude that despite the high temperature of hydrothermal treatment, FeAsS is highly resistant, and the arsenic extraction into the solution is limited. For the most complete transfer of arsenic into the solution, treating finely milled material (10–29 μm) at high temperatures (>523 K) in slightly acidified media (0.05 mol/L H_2_SO_4_) is recommended.

## Figures and Tables

**Table 1:** Particle size analysis.

Size Fraction (μm)	Weight Percent (%)
100+	1.1
71–100	4.8
45–71	10.1
29–45	16.8
10–29	57.2
0–10	10

**Table 2:** Normalized chemical composition of the FeAsS residue before (**1**) and after (**2**) treatment in sodium sulfide solution (wt. %).

№/Component	Cu	Fe	As	S
**1**	44.38	16.97	24.40	14.26
**2**	43.98	18.90	26.30	10.81

**Table 3:** Normalized EDS analysis results (wt.%).

№\Element	Fe	As	S	Cu	Total
Point 1	37.79	44.26	17.95	0	100
Point 2	38.02	44.06	17.92	0	100
Point 3	37.02	45.30	17.68	0	100
Point 4	35.28	47.33	17.39	0	100
Point 5	37.91	45.69	16.40	0	100
Point 6	35.00	46.64	18.36	0	100
Point 7	35.14	45.97	18.89	0	100
Point 8	35.09	46.72	17.96	0.24	100
Point 9	27.69	37.89	24.18	10.24	100
Point 10	2.72	1.46	20.97	74.84	100
Point 11	35.46	45.72	18.16	0.67	100
Point 12	30.01	40.36	24.85	4.78	100
Point 13	23.01	34.79	19.21	22.99	100
Point 14	33.77	40.31	18.85	7.07	100
Point 15	28.62	38.21	18.74	14.43	100
Point 16	19.93	31.68	19.08	29.31	100
Point 17	27.51	40.62	18.71	13.16	100
Point 18	26.02	37.19	19.7	17.09	100
Point 19	30.03	45.37	18.43	6.17	100
Point 20	7.04	11.49	19.09	62.38	100
Point 21	6.9	10.29	15.36	67.65	100
Point 22	34.69	43.37	18.25	3.70	100
Point 23	29.52	39.49	18.25	12.09	100
Point 24	32.26	41.33	16.43	9.98	100
Point 25	26.84	36.79	17.16	19.21	100
Point 26	0	0.3	0.46	99.12	100

**Table 4:** The shrinking core model (SCM) Equations.

№	Limiting Step	Equation
A	Diffusion through the product layer	$1-3\cdot {\left(1-X\right)}^{2/3}+2\cdot (1-X)=kt$
B	New shrinking core model	$1/3\cdot \ln\;(1-X)+{((1-X)}^{-1/3}-1)=kt$
C	Surface chemical reactions	$1-{(1-X)}^{1/3}=kt$

k-a chemical constant, X-FeAsS fraction reacted and *t*-the treatment time.

**Table 5:** SCM equation fitting.

№	SCM Equation	R^2^				
		443 K	463 K	483 K	503 K	523 K
A	$1-3\cdot {\left(1-X\right)}^{2/3}+2\cdot (1-X)=kt$	0.7938	0.6274	0.5085	0.3236	0.1373
B	$1/3\cdot \ln\;(1-X)+{((1-X)}^{-1/3}-1)=kt$	0.8036	0.6433	0.5399	0.3934	0.2388
C	$1-{(1-X)}^{1/3}=kt$	0.3384	0.0795	−0.322	−0.6867	−0.1044

**Table 6:** SCM equation fitting for determined sectors.

№	SCM Equation	R^2^				
		443 K	463 K	483 K	503 K	523 K
**Stage 1**						
A	$1-3\cdot {\left(1-X\right)}^{2/3}+2\cdot (1-X)=kt$	0.9463	0.9792	0.9694	0.9816	0.9101
B	$1/3\cdot \ln\;(1-X)+{((1-X)}^{-1/3}-1)=kt$	0.9956	0.9834	0.9833	0.9937	0.9292
C	$1-{(1-X)}^{1/3}=kt$	0.9773	0.9425	0.6445	0.6224	0.4826
**Stage 2**						
A	$1-3\cdot {\left(1-X\right)}^{2/3}+2\cdot (1-X)=kt$	0.9519	0.9518	0.8909	0.9605	0.9814
B	$1/3\cdot \ln\;(1-X)+{((1-X)}^{-1/3}-1)=kt$	0.9831	0.9817	0.9805	0.9765	0.9782
C	$1-{(1-X)}^{1/3}=kt$	0.9568	0.9671	0.8977	0.9649	0.9853

## Data Availability

Data are contained within the article.
